# Correlation of diaphragm thickening fraction and oesophageal pressure swing in non-invasive ventilation of healthy subjects

**DOI:** 10.1186/s12890-024-03096-5

**Published:** 2024-06-21

**Authors:** Simon Lindner, Clara Hoermann, Jan Teichert, Sevil Ziyadova, Julia D. Michels-Zetsche, Benjamin Neetz, Felix J. F. Herth, Daniel Duerschmied, Simone Britsch

**Affiliations:** 1 Department of Cardiology, Angiology, Haemostaseology and Medical Intensive Care, Medical Centre Mannheim, Medical Faculty Mannheim, Heidelberg University, Theodor-Kutzer-Ufer 1-3, 68167 Mannheim, Germany; 2 European Center for AngioScience (ECAS), German Center for Cardiovascular Research (DZHK) partner site Heidelberg/Mannheim, and Centre for Cardiovascular Acute Medicine Mannheim (ZKAM), Medical Centre Mannheim, Heidelberg University, Mannheim, Germany; 3 Department of Pneumology and Critical Care, Translational Lung Research Center Heidelberg (TLRC-H), Member of the German Center for Lung Research (DZL), Thoraxklinik Heidelberg, University of Heidelberg, Heidelberg, Germany

**Keywords:** Diaphragm Thickening Fraction, Ultrasound, Oesophageal pressure, Respiratory effort, Non-invasive ventilation

## Abstract

**Introduction:**

The diaphragm thickening fraction (DTF) may be a valuable tool for estimating respiratory effort in non-invasive ventilation. The primary aim of this physiological study is the investigation of the correlation of DTF with oesophageal pressure swings (ΔP_oes_). A secondary aim is to assess the discriminatory capacity of the index tests for different exercise loads.

**Methods:**

Healthy volunteers underwent spontaneous breathing and non-invasive ventilation with a sequence of different respirator settings. The first sequence was carried out at rest. The same sequence was repeated twice, with additional ergometry of 25 and 50 Watts, respectively. DTF and ΔP_oes_ were measured during each ventilation configuration.

**Results:**

23 individuals agreed to participate. DTF was moderately correlated with ΔP_oes_ (repeated measures correlation ρ = 0.410, *p* < 0.001). Both ΔP_oes_ and DTF increased consistently with exercise loading in every ventilation configuration, however ΔP_oes_ showed greater discriminatory capacity.

**Conclusion:**

DTF was moderately correlated with ΔP_oes_ and could discriminate reasonably between exercise loads in a small cohort of non-invasively ventilated healthy subjects. While it may not accurately reflect the absolute respiratory effort, DTF might help titrating individual non-invasive respiratory support. Further investigations are needed to test this hypothesis.

**Trial Registration:**

This study was not prospectively registered.

**Supplementary Information:**

The online version contains supplementary material available at 10.1186/s12890-024-03096-5.

## Introduction

Among the respiratory muscles, the diaphragm plays the central role [1, 2]. Its imaging by sonography through an intercostal window in the zone of apposition is a readily available tool. Measuring the expiratory and inspiratory thickness enables the calculation of the diaphragm thickening fraction (DTF) [3]. DTF might be a useful indicator of respiratory effort [4–6]. This is supported by the high correlation between DTF and the pressure achieved during maximum inspiratory efforts [7] and the oesophageal and transdiaphragmatic pressure time products [8, 9]. In acute respiratory failure, quantifying respiratory effort using DTF might be valuable to optimise therapies [8], and its predictive capabilities for failure of non-invasive ventilation have been suggested [10, 11]. Whereas oesophageal or gastral manometry, electromyography and other intricate tools are probably more useful than DTF in mechanical ventilation [12, 13], they are difficult to apply in the context of non-invasive ventilation. Non-invasive respiratory support may be needed not only to increase oxygen delivery, but also to unload ventilatory muscles and reduce respiratory effort through assisted or controlled ventilation. The assessment of DTF in this context yields the potential to monitor and titrate non-invasive respiratory support, as it is one of few practicable ways to approximate respiratory effort beyond clinical impression alone. Oesophageal and gastral manometry may be used [14], however, additional equipment and expertise are needed for this method. Furthermore, the nasogastric probe that is required for this method likely introduces air leak, which could severely impair the efficacy of non-invasive ventilation. We designed this physiological study to compare DTF and oesophageal pressure swings concerning their correlation and their individual capability to discriminate between different respiratory efforts introduced by exercise load.

## Materials and methods

### Study design

This is a physiological study in healthy volunteers.

### Test methods

Voluntary participants were seated on a semi-recumbent bicycle ergometer with 45-degree incline of the torso. There are, to our knowledge, no studies on the reliability of DTF in this position. As it is known that posture influences diaphragm mobility [15, 16] and a semi-seated position is usually used for patients receiving non-invasive ventilation, we thus opted for this experimental setup. A mask for non-invasive respiratory support that covers mouth and nose was used. The experiment was conducted with 3 repeated sequences of different settings of non-invasive ventilation. The first sequence was in resting condition, while in the second and third, ergometry was performed with 40 rpm and loads of 25 and 50 Watts, respectively. During each block, a sequence of 5 phases was performed: The first phase assessed baseline values. Thus, the mask was fastened, but not connected to a ventilator. The second phase was designed to discriminate the impact of the airway resistance introduced by the respiratory circuit, i.e., ventilator, tubing and heat and moisture exchange (HME) filter. The mask was connected to a GE CARESCAPE™ R860 ventilator (GE Healthcare, Chicago, Illinois, USA), with PEEP and inspiratory support pressure set to 0 mbar. In the third phase, PEEP was set to 5 mbar (i.e., CPAP). For the fourth and fifth phase, an inspiratory support pressure of 5 and 10 mbar was added, respectively. Measurements were performed after subjects had adapted to the new setup and were breathing uniformly.

For the measurement of oesophageal pressures, a nasogastric single-balloon catheter was inserted as previously described [17]. To facilitate insertion, xylocaine spray was used for local anaesthesia of the nose and pharynx and the catheter was lubricated with xylocaine gel. After placement, the balloon was inflated with 6 ml of air, followed by aspiration of 2 ml, resulting in a balloon filling volume of 4 ml. Placement below the diaphragm was confirmed by pressure increase during a prompted inspiratory effort. The catheter was then retracted until cardiac oscillations were maximal and negative inspiratory pressure swings confirmed supradiaphragmatic placement. The oesophageal pressure swing (ΔP_oes_) was calculated as the end-expiratory P_oes_ minus the nadir of the inspiratory P_oes_ deflection [18].

Diaphragm ultrasound was acquired at the zone of apposition in the right mid-axillary line. B-Mode images of end-inspiratory and end-expiratory diaphragm thickness (DT) were measured in each cycle. DTF was calculated as:

$$DTF\, = \,100\,*\,\frac{\left( {\left( {end{\rm{ - }}inspiratory\,DT} \right) - \left( {end{\rm{ - }}expiratory\,DT} \right)} \right)}{\left( {end{\rm{ - }}expiratory\,DT} \right)}$$

Two uniform respiratory cycles were used for measurement and calculation of DTF and ΔP_oes_. Mean values were used for analysis. The investigators were not blinded to the study phases. Respiratory rates were recorded for all phases. Minute ventilation and air leak measured by the ventilator was recorded for phases 2 to 5. Tidal volume was calculated by division of minute ventilation by respiratory rate.

### Analysis

Qualitative variables are given in absolute numbers and group related percentages. Metric variables are given as medians and interquartile range (IQR). R software version 4.3.1 with the rmcorr-package [19] version 0.5.4 was used to calculate the repeated measures correlation of DTF and ΔP_oes_. Differences of measurements between exercise loads were compared with the related-samples Friedman’s two-way analysis of variance by ranks using IBM® SPSS ® Statistics Version 28.0.1.0 (IBM, Armonk, NY).

## Results

### Participants and baseline values

25 healthy volunteers agreed to participate in this study. 2 subjects had to be excluded as placement of the nasogastric balloon catheter was aborted due to severe faucial reflex, both were male. 23 participants underwent all study procedures according to protocol. Median age was 28 (IQR 23–36) years, median body mass index 23 (IQR 22.6–24.9) kg/m^2^ and 12 (52%) were female. Median end-expiratory diaphragm thickness during resting conditions and breathing without connection to the ventilator was 1.7 mm (IQR 1.4–1.8 mm) and DTF was 22% (IQR 17–32%). Median end-expiratory oesophageal pressure was 9 mbar (IQR 5–10 mbar) and ΔP_oes_ was 4 mbar (IQR 3–5 mbar). All baseline values are displayed in [Table 1].

**Table 1:** Baseline characteristics

Total number of participants	23
Age [years] (IQR)	28 (23–36)
Female sex, n (%)	12 (52)
Height [cm] (IQR)	176 (165–182)
Weight [kg] (IQR)	72 (62–72)
BMI [kg/cm^2^] (IQR)	23 (23–25)
End-expiratory diaphragm thickness [mm] (IQR)	1.7 (1.4–1.8)
End-expiratory oesophageal pressure [mbar] (IQR)	9 (5–10)
Diaphragm thickening fraction [%] (IQR)	22 (17–32)
Oesophageal pressure swing [mbar] (IQR)	4 (3–5)

IQR = Interquartile range

### Correlation of DTF and ΔP_oes_

Every participant underwent 3 study sequences, each consisting of 5 identical ventilation setups. A set of measurements was acquired during every constellation. Thus, 345 measurements of DTF and corresponding ΔP_oes_ were recorded. Repeated measures correlation was calculated as ρ = 0.410 (95% CI 0.315–0.497, *p* < 0.001).

### Discrimination between exercise loads

**Fig. 1:**
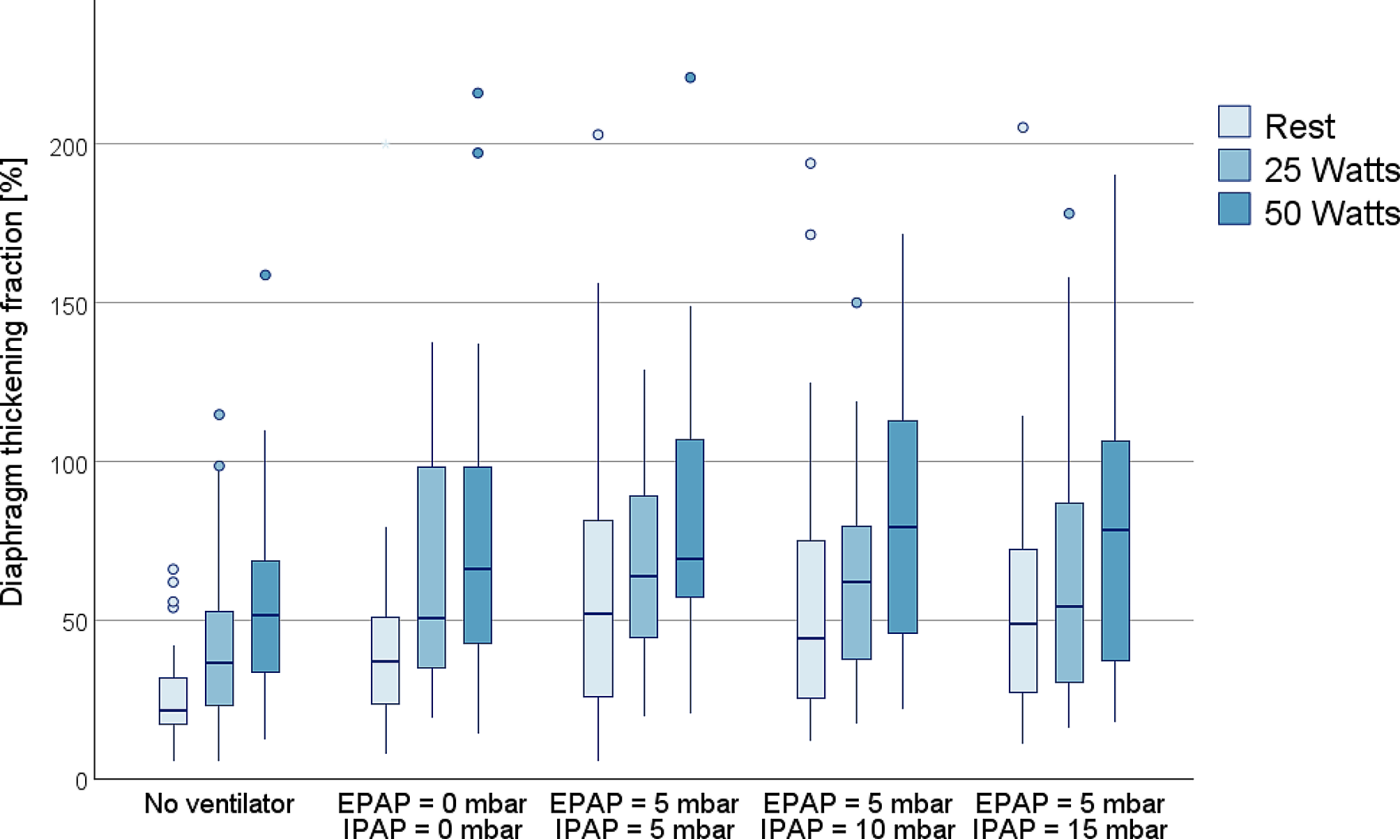
Boxplots of diaphragm thickening fraction measurements according to exercise load and ventilator setting Circles mark data outliers (3rd quartile + 1.5*interquartile range or 1st quartile – 1.5*interquartile range) Asterisks mark extreme outliers (3rd quartile + 3*interquartile range or 1st quartile – 3*interquartile range) Between-group differences: No ventilator *p* = 0.005, EPAP 0 mbar/IPAP 0 mbar *p* = 0.003, EPAP 5 mbar/IPAP 5 mbar *p* = 0.054, EPAP 5 mbar/IPAP 10 mbar *p* = 0.042, EPAP 5 mbar/IPAP 15 mbar *p* = 0.054

**Fig. 2:**
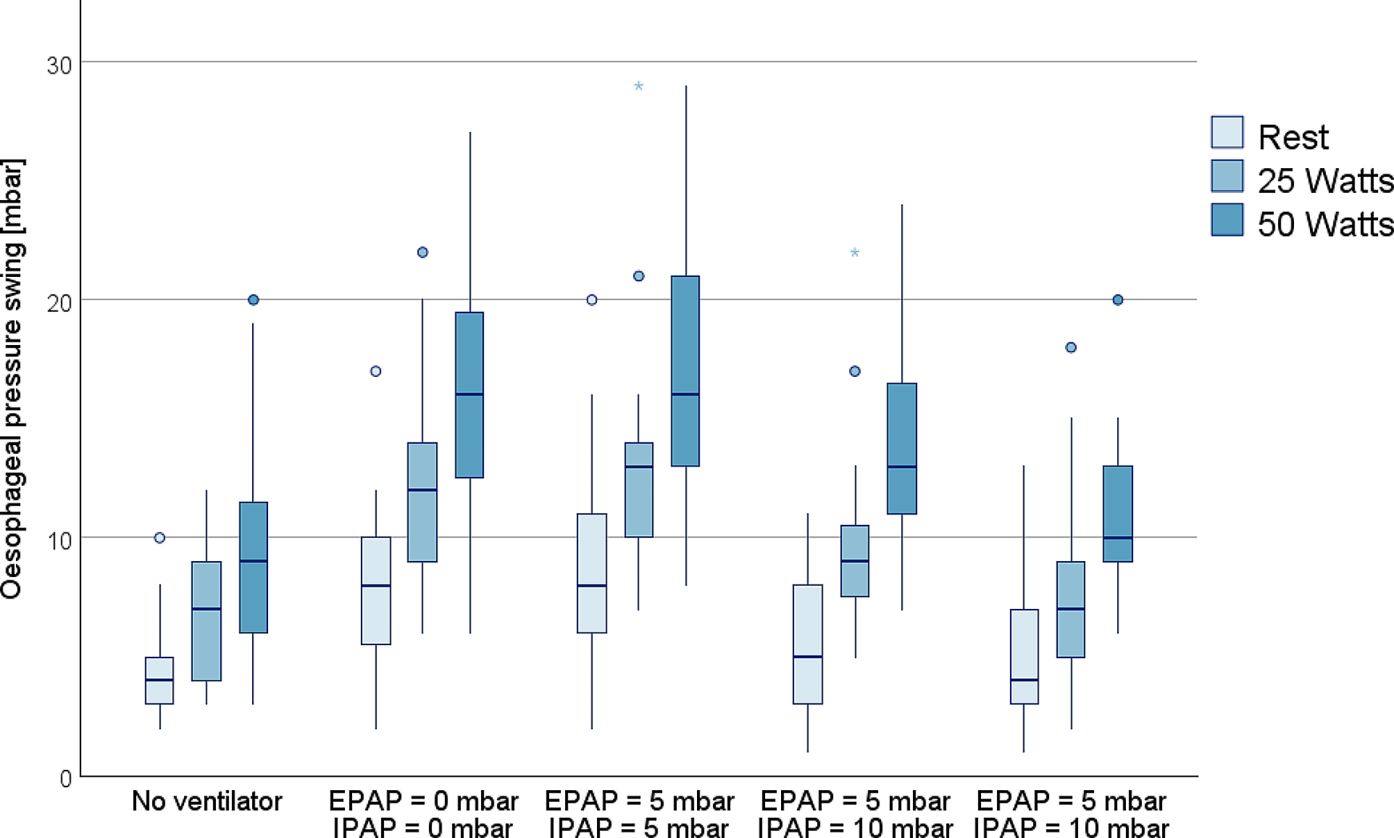
Boxplots of diaphragm thickening fraction measurements according to exercise load and ventilator setting Circles mark data outliers (3rd quartile + 1.5*interquartile range or 1st quartile – 1.5*interquartile range) Asterisks mark extreme outliers (3rd quartile + 3*interquartile range or 1st quartile – 3*interquartile range) Between-group differences: No ventilator *p* < 0.001, EPAP 0 mbar/IPAP 0 mbar *p* < 0.001, EPAP 5 mbar/IPAP 5 mbar *p* < 0.001, EPAP 5 mbar/IPAP 10 mbar *p* < 0.001, EPAP 5 mbar/IPAP 15 mbar *p* < 0.001

In every ventilatory setup, both DTF and ΔP_oes_ values increased with exercise loading. **[**Figs. 1 and 2**]** show the boxplots for ventilatory setup and exercise load related ΔP_oes_ and DTF measurements.

Although measures were taken to minimise air leakage, e.g. fastening of the mask, rearrangement of the mask exit of the nasogastric tube and the use of seal-pads, it could not be avoided in most cases. Air leakage was increasing throughout the study sequences, with 10% (IQR 0–10%) when connected to the ventilator circuit, 20% (IQR 10–30%) during CPAP of 5 mbar and with additional 5 mbar inspiratory pressure support, as well as 20% (IQR 10–40%) with 10 mbar pressure support. All air leaks were attributable to the nasogastric catheter. All variables recorded during each experimental setup are provided as [Supplementary Table. 1].

## Discussion

In this physiological study, we could demonstrate a moderate correlation of DTF with ΔP_oes_ in healthy individuals during spontaneous breathing and different settings of non-invasive ventilation. Our findings are similar to the association of DTF with changes in transdiaphragmatic pressure and transdiaphragmatic pressure-time product (PTP_di_) found in spontaneously breathing healthy subjects by Poulard et al. [13]. Vivier et al. reported a stronger correlation of DTF and the PTP_di_ in a cohort of non-invasively ventilated patients after extubation [7]. Several explanations are worth to be discussed. Firstly, the PTP_di_ is a marker which is solely attributable to force generated by the diaphragm. The ΔP_oes_ is influenced by the force generated by the diaphragm as well as the extradiaphragmatic inspiratory muscles like the external and parasternal intercostals and accessory inspiratory muscles, possibly rendering the correlation weaker. Secondly the abdominal muscles are recruited under exercise [20]. The concomitant rise in abdominal pressure is transmitted into the pleural space, which elevates the end-expiratory P_oes_ and thereby the ΔP_oes_ [21]. Since the associated increase in ΔP_oes_ in this case is not attributable to the diaphragm, this could weaken the correlation between DTF and ΔP_oes_. The inter-individual differences in the recruitment of diaphragmatic and extra-diaphragmatic respiratory muscles could also explain the variable and sometimes slightly negative correlations between DTF and ΔP_oes_. Furthermore, DTF is not inherently an index of effort, but rather an indicator of muscle activity. Therefore, it may not closely correlate with ΔP_oes_, especially in healthy volunteers.

The secondary aim was to explore the capabilities of the index tests to differentiate between resting condition and exercise loads of 25 and 50 Watts. Although ΔP_oes_ showed a clearer distinction between exercise loads in all experimental setups, DTF performed reasonably well in this small sample size. Our findings are less clear than reports by Umbrello et al., where they found similar distinction abilities of ΔP_oes_ and DTF between pressure support levels in invasively ventilated patients [9]. Bicycle ergometry could have impaired DTF measurements in our setup, as measurements were taken during active pedalling.

In spite of these findings, DTF might still be preferable to oesophageal or gastric pressure measurements for titrating non-invasive respiratory support, as the nasogastric probe is associated with discomfort for patients that have to be alert, in contrast to sedated patients undergoing invasive ventilation. In our cohort, 2 subjects had to be excluded as placement of the nasogastric probe was aborted due to a distinct faucial reflex. Moreover, the probe often prevents complete sealing of the mask and thus leads to air leakage. Air leakage is known to impair the effectiveness of non-invasive ventilation through loss of airway pressure and delayed cycling [22, 23]. In this experiment, we were unable to eliminate air leakage, despite adequate measures for seal optimisation were taken. In our opinion, this aspect highlights one clear advantage of DTF in this context. Additionally, ultrasound is a readily available tool in most intensive care units.

Although DTF may not be able to accurately assess absolute values for breathing effort, we could demonstrate its capability to detect relative changes in effort induced by exercise. We could also recreate a previous investigation where we found a decrease of DTF through introduction of pressure support [24] similar to reports by Vivier et al. [8]. These findings indicate its potential for titration of non-invasive respiratory support, but further studies are needed to delineate the use of DTF in a clinical environment.

### Limitations of this study

The main limitation of this physiological study is that measurements were performed in healthy subjects of normal weight and the relatively small sample size. In addition, we did not measure the gold standard for respiratory effort, which is the pressure time product of the respiratory muscle pressure measured over a 1 min period (PTP_mus_) [18], as DTF and ΔP_oes_ both represent parameters that do not take time into account. We believe that the study results are only conditionally influenced by this, as ΔP_oes_ was shown to be an adequate estimate of inspiratory effort in two studies [9, 25]. The incorporation of respiratory rate may, however, be relevant for the clinical application of DTF [26]. Investigators were not blinded to the experimental setup and sequence. No direct conclusions should be drawn for clinical practice and for the treatment of patients.

## Conclusion

DTF was moderately correlated with ΔP_oes_ and could discriminate reasonably between exercise loads in a small cohort of non-invasively ventilated healthy subjects. While it may not accurately reflect the absolute respiratory effort, DTF might indicate relative changes and could thus potentially help titrating individual non-invasive respiratory support. Further investigations are needed to test this hypothesis.

### Electronic supplementary material

Below is the link to the electronic supplementary material.

Supplementary Material 1

## Data Availability

The data that support the findings of this study are not publicly available due to privacy reasons. Anonymised data are available from the corresponding author upon reasonable request.
