# Supplementary material for: Correlation of diaphragm thickening fraction and oesophageal pressure swing in non-invasive ventilation of healthy subjects
Source: BMC Pulm Med. 2024 Jun 21;24:289. doi: 10.1186/s12890-024-03096-5 (PMC11191247; doi:10.1186/s12890-024-03096-5)
Supplement: Supplementary file 1 — Supplementary Material 1 [file 12890_2024_3096_MOESM1_ESM.docx]

| Supplementary Table 1 All measured variables according to phase and exercise load | | | | | | | | | | | | | | | | | | |
| --- | --- | --- | --- | --- | --- | --- | --- | --- | --- | --- | --- | --- | --- | --- | --- | --- | --- | --- |
| **Ventilator setting** | **EPAP** | **none** | | | **0** | | | | **5** | | | **5** | | | | **5** | | |
|  | **IPAP** | **none** | | | **0** | | | | **5** | | | **10** | | | | **15** | | |
| **Exercise load** | | **Rest** | **25 W** | **50 W** | **Rest** | **25 W** | | **50 W** | **Rest** | **25 W** | **50 W** | **Rest** | | **25 W** | **50 W** | **Rest** | **25 W** | **50 W** |
| Expiratory diaphragm thickness [mm]  (IQR) | | 1.7  (1.4-1.8) | 1.7  (1.5-1.9) | 1.7  (1.4-1.9) | 1.7  (1.5-2.0) | 1.8  (1.5-2.0) | | 1.8  (1.6-2.1) | 1.8  (1.6-2.1) | 1.9  (1.6-2.0) | 1.9  (1.7-2.2) | 1.8  (1.5-2.1) | | 1.8  (1.7-2.2) | 1.8  (1.5-2.1) | 1.8  (1.6-2.0) | 1.8  (1.6-2.1) | 1.8  (1.6-2.1) |
| Inspiratory diaphragm thickness [mm]  (IQR) | | 2.0  (1.8-2.3) | 2.5  (2.0-2.9) | 2.6  (2.3-2.8) | 2.5  (2.1-3.0) | 2.7  (2.5-3.2) | | 3.2  (2.7-3.7) | 2.9  (2.5-3.4) | 2.9  (2.6-3.7) | 3.1  (2.9-4.3) | 2.8  (2.2-3.3) | | 2.9  (2.4-3.6) | 3.2  (2.9-4.0) | 2.7  (2.3-3.4) | 2.7  (2.2-3.8) | 3.4  (2.6-3.8) |
| Diaphragm thickening fraction [%]  (IQR) | | 22  (17-32) | 36  (23-56) | 52  (33-70) | 37  (23-52) | 50  (33-100) | | 66  (43-99) | 52  (24-82) | 64  (43-89) | 69  (57-116) | 44  (24-79) | | 62  (34-84) | 79  (45-114) | 49  (27-76) | 54  (29-88) | 78  (32-116) |
| Expiratory oesophageal pressure [mbar]  (IQR) | | 9  (5-10) | 12  (8-15) | 12  (7-16) | 9  (4-12) | 12  (9-16) | | 13  (9-17) | 9  (6-15) | 13  (11-16) | 14  (11-18) | 9  (7-12) | | 13  (10-18) | 12  (11-18) | 11  (8-13) | 13  (9-18) | 13  (10-16) |
| Inspiratory oesophageal pressure [mbar]  (IQR) | | 3  (0-6) | 5  (0-9) | 1  (-3-5) | 1  (-3-5) | 0  (-3-4) | | -4  (-7-(-1)) | 1  (-2-7) | -1  (-3-2) | -4  (-7-0) | 3  (0-7) | | 4  (0-7) | -1  (-5-3) | 6  (2-8) | 5  (2-9) | 1  (0-6) |
| Oesophageal pressure swing [mbar]  (IQR) | | 4  (3-5) | 7  (4-9) | 9  (6-12) | 8  (5-10) | 12  (8-14) | | 16  (12-20) | 8  (6-11) | 13  (10-17) | 16  (13-22) | 5  (3-8) | | 9  (7-11) | 13  (11-17) | 4  (3-7) | 7  (5-9) | 10  (9-14) |
| Tidal volume [l]  (IQR) | | - | - | - | 0.7  (0.5-0.9) | 1.0  (0.8-1.3) | | 1.4  (1.2-1.6) | 1.0  (0.7-1.2) | 1.2  (1.0-1.4) | 1.6  (1.3-1.7) | 1.1  (0.8-1.2) | | 1.4  (1.1-1.6) | 1.6  (1.4-1.9) | 1.2  (1.0-1.5) | 1.4  (1.3-1.7) | 1.7  (1.3-2.1) |
| Respiratory rate [bpm]  (IQR) | | 15  (12-16) | 16  (12-19) | 15  (12-22) | 12  (11-14) | 14  (11-16) | | 13  (12-16) | 11  (9-12) | 12  (10-14) | 14  (12-16) | 12  (10-13) | | 12  (11-14) | 13  (11-16) | 11  (10-13) | 12  (10-16) | 13  (11-16) |
| Minute ventilation [l/min]  (IQR) | | - | - | - | 9  (7-10) | 14  (11-16) | | 19  (17-21) | 10  (8-13) | 15  (13-17) | 21  (19-23) | 12  (10-14) | | 17  (13-19) | 22  (20-26) | 14  (13-17) | 18  (16-21) | 22  (20-26) |
| Air leak [%]  (IQR) | | - | - | - | 10  (0-10) | 10  (0-10) | | 5  (0-10) | 30  (0-30) | 20  (10-30) | 10  (10-20) | 30  (10-50) | | 20  (10-40) | 20  (10-20) | 40  (10-50) | 20  (10-40) | 20  (10-30) |
| EPAP = Expiratory positive airway pressure; IPAP = Inspiratory positive airway pressure; IQR = Interquartile range | | | | | | | | | | | | | | | | | | |
|  |  | | | | | |  | | | | | |  | | | | | |
